# Pathological tau drives ectopic nuclear speckle scaffold protein SRRM2 accumulation in neuron cytoplasm in Alzheimer’s disease

**DOI:** 10.1186/s40478-021-01219-1

**Published:** 2021-06-29

**Authors:** Pamela J. McMillan, Timothy J. Strovas, Misa Baum, Brooke K. Mitchell, Randall J. Eck, Nzinga Hendricks, Jeanna M. Wheeler, Caitlin S. Latimer, C. Dirk Keene, Brian C. Kraemer

**Affiliations:** 1 Geriatrics Research Education and Clinical Center, Veterans Affairs Puget Sound Health Care System, S182, 1660 South Columbian Way, Seattle, WA 98108 USA; 2 Department of Psychiatry and Behavioral Sciences, University of Washington, Seattle, WA 98195 USA; 3 Graduate Program in Neuroscience, University of Washington, Seattle, WA 98195 USA; 4 Department of Laboratory Medicine and Pathology, University of Washington, Seattle, WA 98195 USA; 5 Division of Gerontology and Geriatric Medicine, Department of Medicine, University of Washington, Seattle, WA 98104 USA

**Keywords:** Nuclear speckles, Alzheimer’s disease, MSUT2, PABPN1, Tau, MapT tauopathy PS19, SRRM2, SC-35, ZC3H14

## Abstract

Several conserved nuclear RNA binding proteins (*sut-1, sut-2,* and *parn-2*) control tau aggregation and toxicity in *C. elegans*, mice, and human cells. MSUT2 protein normally resides in nuclear speckles, membraneless organelles composed of phase-separated RNAs and RNA-binding proteins that mediate critical steps in mRNA processing including mRNA splicing. We used human pathological tissue and transgenic mice to identify Alzheimer’s disease-specific cellular changes related to nuclear speckles. We observed that nuclear speckle constituent scaffold protein SRRM2 is mislocalized and accumulates in cytoplasmic lesions in AD brain tissue. Furthermore, progression of tauopathy in transgenic mice is accompanied by increasing mislocalization of SRRM2 from the neuronal nucleus to the soma. In AD brain tissue, SRRM2 mislocalization associates with increased severity of pathological tau deposition. These findings suggest potential mechanisms by which pathological tau impacts nuclear speckle function in diverse organisms ranging from *C. elegans* to mice to humans. Future translational studies aimed at restoring nuclear speckle homeostasis may provide novel candidate therapeutic targets for pharmacological intervention.

**Supplementary Information:**

The online version contains supplementary material available at 10.1186/s40478-021-01219-1.

## Introduction

Amyloid beta (Aβ) plaques and neurofibrillary tangles (NFTs) containing aggregates of hyperphosphorylated tau represent the diagnostic pathological lesions present in Alzheimer’s disease (AD) brains. The mechanism of interaction between Aβ plaques and NFTs remains incompletely understood, but both contribute to neurodegeneration in AD. The discovery of mutations causing FTLD-tau in the human gene encoding tau protein has demonstrated amyloid independent tau neurotoxicity [1–3] and tangles, rather than plaques, correlate most closely with the severity of dementia in AD [4]. These observations support the need for further exploration of the mechanistic underpinnings of both Aβ- and tau-mediated neurodegeneration in AD. Here we focus on disease mechanisms relating pathological tau to nuclear dysfunction.

Recent work has implicated nuclear dysfunction of RNA-binding proteins as a driver of neurodegeneration in model organisms and cellular systems [5–8]. RNA acts as a potent initiator of tau aggregation in vitro and can decorate fibrillar tau after aggregation [9–11]. Most recently, tau has been shown to form aggregates with an RNA protein assembly in some cellular contexts [7]. The recruitment of RNA to tau aggregates may drive splicing defects or disrupt other nuclear functions related to RNA [5–7, 12]. These observations suggest the possibility of tauopathy-driven RNA-mediated cellular dysfunction, but these phenomena have not been investigated in the context of AD pathology. Here, we begin to address the role of RNA binding protein involvement with pathological tau in AD, guided by findings from molecular genetic investigations in model systems.

In our previous genetic studies of tauopathy in C. elegans, we identified multiple genes whose loss of function suppress tauopathy neurodegenerative phenotypes (suppressor of tauopathy or sut genes). The largest group of *sut* genes isolated encode proteins residing in nuclear speckles, which suggests modulating nuclear speckle function can ameliorate tauopathy. Conserved *sut* genes may also play a role in tau mediated neurodegeneration in mammals. The first tauopathy suppressor isolated, *sut-1*, exhibits strong suppression of tauopathy phenotypes; SUT-1 protein localizes to nuclear speckles [13] and is thought to mediate spliceosome recycling [14]. The *parn-2* gene is another recently isolated partial tau suppressor gene encoding a protein known to reside in nuclear speckles with poly(A) RNase activity [15].

To determine whether conserved *sut* genes may play a role in tau mediated neurodegeneration in mammals, we have explored the translational potential of *sut-2.* We first identified *sut-2* as a suppressor of tau-induced neurodegenerative defects in *C. elegans* [16]. The *sut-2* gene encodes a zinc finger protein with a single conserved homolog in diverse species ranging from yeast to humans. The mammalian SUT-2 protein (MSUT2) binds to RNA and localizes to nuclear speckles, membraneless organelles coordinating mRNA processing in the nucleus [17, 18]. Knockout of the *MSUT2* gene in mice results in animals that develop appropriately and appear healthy, live a normal lifespan, and exhibit normal neurological function including learning and memory [19]. We have shown that *MSUT2* KO in mice can suppress tauopathy phenotypes in a transgenic mouse model expressing human tau protein. PS19 tau transgenic mice exhibit early stages of tauopathy by 3 months of age that progress to neurofibrillary degeneration by 8 months. These mice exhibit pathological tau and cognitive dysfunction resembling the changes observed in human AD patients or those with other tauopathy disorders. When combined with the PS19 transgene, *MSUT2* gene knockout ameliorates a variety of pathological tau lesions. For instance, *MSUT2* KO decreases accumulation of phosphorylated tau (pTau) lesions as represented by immunostaining with AT180, an early marker of pathological tau [20]. Likewise, *MSUT2* KO decreased pre-tangle conformations of tau, critical for the eventual formation of mature NFTs [21]. The deposition of tangles is accompanied by obvious neurodegeneration in PS19 Tg mice and *MSUT2* KO reduces the tau-mediated loss of neurons in the hippocampus [19]. The amelioration of neurodegeneration is also accompanied by a reduction in astrocytosis as indicated by GFAP staining [19]. We also found *MSUT2* KO mice exhibit robust amelioration of tauopathy-related behavioral phenotypes, including a restoration of spatial memory function as detected by the Barnes maze test.

Given the above findings demonstrating MSUT2 involvement in determining susceptibility to pathological tau, combined with its role as a nuclear speckle-localized RNA-binding protein, we have begun to explore the role of RNA-binding protein-mediated pathological consequences of tauopathy in AD. In this study, we investigated another nuclear speckle protein, SRRM2, which is a core nuclear speckle scaffold protein required for the formation of nuclear speckles. Recent studies indicate SRRM2 abnormality in AD cellular and animal models [7, 22]. Guided by findings from molecular genetic investigations in model systems, the current study examines the impact of pathological tau on SRRM2 protein in the brains of AD patients and mouse models of tauopathy.

## Materials and methods

### Brain tissue from AD and control cases

Samples of postmortem brain tissue were obtained from the University of Washington (UW) Alzheimer’s Disease Research Center (ADRC) and the Adult Changes in Thought (ACT) Study via the UW BioRepository and Integrated Neuropathology Laboratory. Informed consent for research brain donation was obtained from the legal next of kin according to protocols approved by the UW Institutional Review Board. AD cases (n = 29) were selected based on a clinical diagnosis of dementia and autopsy-confirmed AD neuropathologic change (ADNC) sufficient to explain dementia. Control postmortem brain samples (n = 7) were from age-matched cognitively normal research participants with autopsy-confirmed absent-low levels of ADNC. Donor brains were fixed in 10% neutral buffered formalin for at least two weeks, coronally sliced, and samples of prefrontal cortex, hippocampus, amygdala and cerebellum processed and embedded in paraffin, and sectioned at 5 micron thickness according to routine protocols. Tissue was subjected to histological and neuropathological analysis as described below (Table 1).

**Table 1:** Case details

Case #	Dx (NP verified	Age at death	Sex	Age at onset	Duration (years)	PMI (hrs)	NIA-AA B score	NIA-AA C score
1	AD	76	M	65	11	4:30	3	3
2	AD	82	F	55	27	8:00	3	3
3	AD	60	F	54	6	7:30	2	2
4	AD	82	M	76	6	8:00	2	3
5	AD	80	F	69	11	6:00	3	3
6	AD	71	F	60	11	5:00	3	3
7	AD	81	F	71	10	4:00	3	3
8	AD	70	F	62	8	5:00	3	3
9	AD	68	M	58	10	9:25	3	3
10	AD	77	M	57	20	7:25	3	3
11	AD	84	F	76	8	4:47	3	3
12	AD	93	F	84	9	6:30	3	1
13	AD	62	M	55	7	4:32	3	3
14	AD	88	F	77	11	5:40	3	3
15	AD	78	M	72	6	7:00	3	3
16	AD	86	F	76	10	8:32	3	3
17	AD	75	M	64	11	4:30	3	3
18	AD	90	F	76	14	6:23	3	3
19	AD	89	F	82	7	5:23	3	2
20	AD	77	M	71	6	6:32	3	3
21	AD	78	F	69	9	2:20	3	2
22	AD	94	M	91	3	3:05	3	3
23	AD	86	M	80	6	2:40	3	3
24	AD	77	M	63	14	5:20	2	3
25	AD	100	F	82	18	3:08	3	2
26	AD	91	M	82	9	7:00	3	2
27	AD	94	M	82	12	4:25	3	3
28	AD	86	M	81	5	4:25	3	3
29	AD	89	M	86	3	6:25	3	3
30	CNT	92	M	NA	NA	4:20	1	1
31	CNT	81	M	NA	NA	5:45	1	1
32	CNT	78	F	NA	NA	6:00	1	1
33	CNT	95	M	NA	NA	4:00	1	1
34	CNT	91	F	NA	NA	6:00	2	1
35	CNT	78	M	NA	NA	5:35	2	1
36	CNT	79	F	NA	NA	7:00	1	1

### Mouse experiments

All mouse experiments were reviewed and approved by the VA Puget Sound Health Care System Institutional Animal Care and Use Committee (IACUC) and conducted in an American Association for Accreditation of Laboratory Animal Care (AAALAC)-accredited animal research facility. The PS19 tau transgenic mouse model expressing human P301S mutant human tau was used in this study. This mouse model is well characterized and has a highly progressive tauopathy related phenotype. In addition, a milder mouse model of tauopathy driven by wild type human tau expression (Tau4Rtg2652) was also examined. This mouse line exhibits early-stage tau pathology, including phosphorylated tau, but not neurofibrillary degeneration [23]. PS19 mice (15 three month, 7 at seven months of age and 16 at nine months of age), Tau4Rtg2652 mice (8 at 4 months of age) and WT mice (5 at nine months of age) were anesthetized and fixed by transcardial perfusion with 4% paraformaldehyde. Brains were removed and paraffin embedded for sectioning. Coronal sections (9 microns) were prepared and stored at 4 °C until use.

### Tissue Immunohistochemistry and staining

Human and mouse brain sections were deparaffinized, rehydrated through alcohols, and processed through antigen retrieval steps consisting of heat pretreatment in citrate buffer by either microwave or autoclave per antibody-specific protocols. Sections were treated for endogenous peroxidases with 3% hydrogen peroxide in PBS (pH 7.4), blocked in 5% non-fat milk in PBS, and incubated with primary antibodies overnight at 4 °C (see Table 2). Biotinylated secondary antibody was applied for 45 min at room temperature. Finally, sections were incubated in an avidin–biotin complex with streptavidin-HRP (Vector’s Vectastain Elite ABC-HRP kit, Burlingame, CA) and the reaction product was visualized with 0.05% diaminobenzidine (DAB)/0.01% hydrogen peroxide in PBS. Negative controls consisted of full protocol except primary antibody. The presence of neurofibrillary tangles was assessed by Gallyas silver staining using standard methods. Digital images were obtained using a Leica DM6 microscope with a DFC 7000 digital camera (Leica Microsystems, Wetzlar, Germany) and imported into Adobe Photoshop (Adobe Inc, San Jose, CA).

### Statistical analysis

For quantitation in human tissue, immunostained sections were analyzed using the computer image analysis system MicroComputer Imaging Device (MCID; Imaging Research, St. Catherines, Ontario, Canada) and blinded assessment of optical density measurements were obtained relative to the proportional area for AT180 and NeuN immunostaining in frontal cortex grey matter (three separate readings per case) as previously described [19]. For quantitation in mouse brain tissue, the number of neurons with cytoplasmic pSRRM2 inclusions were manually counted across the entire section (two sections per animal). Data were averaged and are represented as means ± SEM. A two-tailed Student’s t test was used to assess differences in staining intensity between experimental groups. Statistical analysis and graphing was performed using the Prism V8.3 software package (GraphPad).

### Immunofluorescence microscopy, co-localization, and proximity ligation studies

Human frontal cortex sections were deparaffinized followed by antigen retrieval as described above. Sections were permeabilized in 0.2% Triton X-100 in PBS, blocked with 2% goat serum in PBS and incubated overnight at 4 °C with primary antibodies to pSRRM2 (mouse mAb clone SC-35, [24] and pS422 (rabbit mAb clone EPR2866) or DAKO anti-tau (rabbit polyclonal anti-tau). For double label immunofluorescence, secondary antibodies AlexaFluor 488 goat anti-rabbit and AlexaFluor 647 goat anti-mouse secondary antibodies (ThermoFisher, Waltham, MA) were applied for 45 min at room temperature and sections were counterstained with 300 nM DAPI before mounting with ProLong Gold Antifade. Images were obtained on a DeltaVision Elite microscope (Cytava Lifesciences, Marlborough, MA) using a 100X oil immersion objective and colocalization analysis was performed with SoftwoRx 6.0 Beta software (Cytiva).

Proximity Ligation assays were performed using Duolink PLA technology as recommended by the manufacturer (Sigma-Aldrich Catalog# DUO92101,St. Louis, MO) using sections prepared through primary antibody incubation as described above. Human brain samples assayed for pSRRM2 and pS422 colocalization were imaged on a Nikon A1R confocal microscope using a 100 × oil immersion objective (Nikon USA, Melville, NY).

### Results

### Phosphorylated nuclear speckle scaffold protein SRRM2 becomes mislocalized in Alzheimer’s disease

**Fig. 1:**
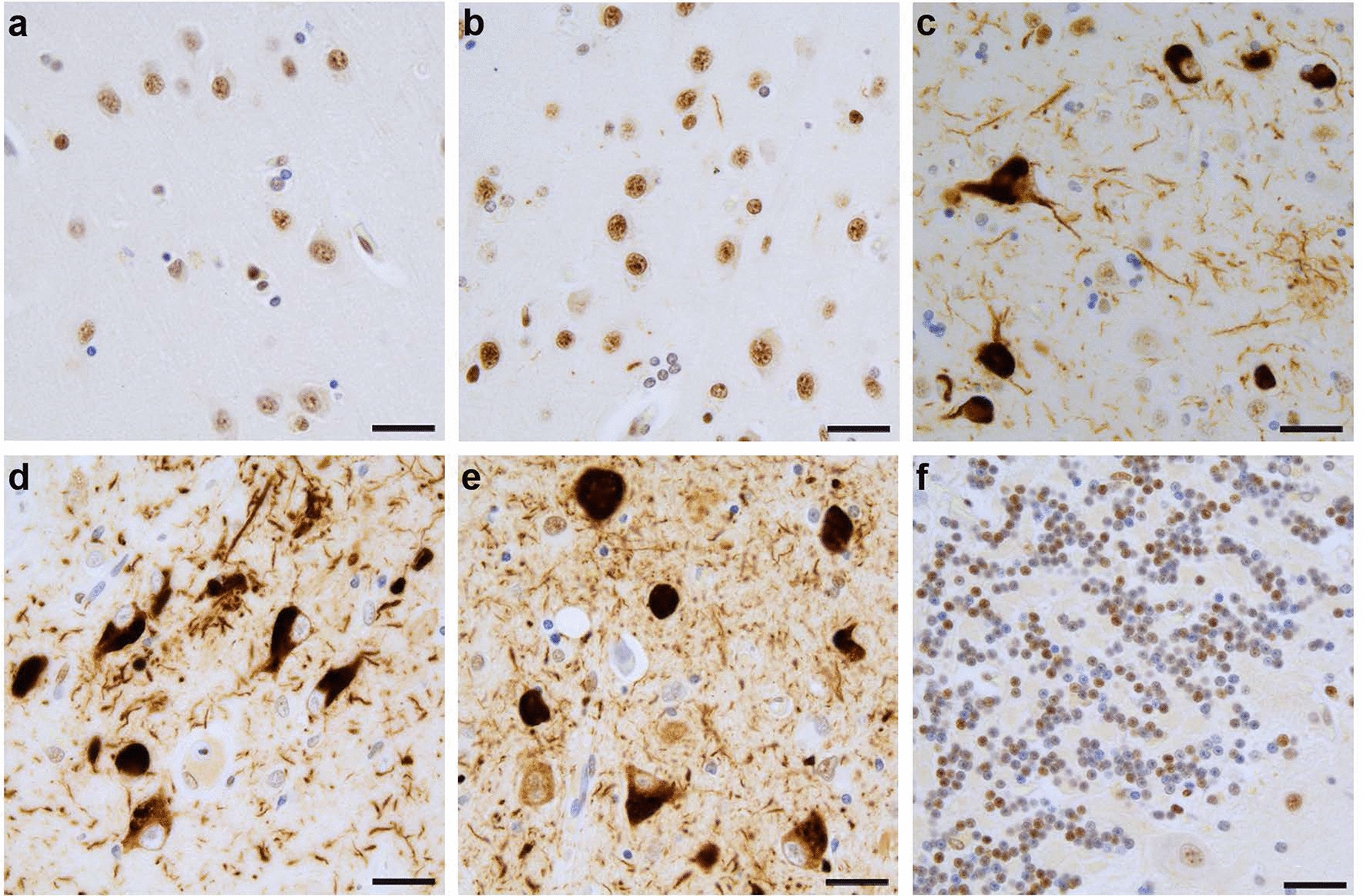
Derangement of nuclear speckle marker pSRRM2 (ab SC-35) in AD. Immunohistochemistry using a pSRRM2 recognizing antibody raised against purified human spliceosomes (clone SC-35, see Table 2). Human pSRRM2 stains nuclear speckles within neurons in the frontal cortex of the normal adult human brain (**a**). A subset of AD donors (30%) also exhibit normal nuclear speckle staining in cortical neurons (**b**), however, the majority of AD donors (72%) exhibit striking mis-localization of pSRRM2 to the cytoplasm in neurons in the frontal cortex (**c**). Additionally, all AD donors exhibited mis-localization of pSRRM2 in the hippocampus (**d**) and amygdala (**e**), brain regions where tauopathy occurs earlier in disease progression. AD donors with aberrant cytoplasmic pSRRM2 in tauopathy rich regions showed normal nuclear pSRRM2 distribution in cerebellar neurons (**f**), which do not typically exhibit any tau pathology. Scale bars, 50 µm

Since *sut-2* and its homolog MSUT2 enable the development of tau pathology and appear to reside in nuclear speckles within the nuclei of neurons [13, 16, 25], we hypothesized that nuclear speckle function may play an important role in the genesis or progression of pathological tau. To further investigate the involvement of nuclear speckles in neurodegenerative tauopathy, we immunostained brain sections from AD cases for the canonical nuclear speckle marker monoclonal antibody (mAb) clone SC-35. The mAb SC-35 was originally raised against purified human spliceosomes and has recently been conclusively demonstrated to recognize a phosphorylated epitope of human SRRM2 (pSRRM2) protein [24, 26]. SRRM2 protein plays a key role in mRNA splicing as a nuclear matrix protein [27, 28] and is a critical nuclear speckle co-scaffolding protein with SON protein [26]. Immunostaining with mAb SC-35 demonstrates a clear disruption of the normal canonical nuclear speckle staining pattern in the frontal cortex of a majority of AD cases, but not in age-matched controls (Fig. 1). The striking mis-localization of SRRM2 appears to be a relocalization of pSRRM2 protein from the nuclear speckles within neuronal nuclei to the soma, with pSRRM2 accumulating in the cytoplasm. This abnormal pSRRM2 staining pattern occurs in the frontal cortex in 20 out of 29 AD cases. Furthermore, this cytoplasmic distribution of pSRRM2 was detected in all 29 AD cases in the hippocampus and amygdala, regions known to accumulate abundant tau pathology at an earlier stage in the disease process. In contrast, we did not observe pSRRM2 mis-localization in the cerebellum, a brain region generally spared from neurodegeneration and pathological tau in AD; rather we observed a normal pattern clearly marking nuclear speckles. Taken together, the pattern of neurons exhibiting pSRRM2 mis-localization parallels the pattern of pathological neurofibrillary tangle deposition in AD brain.

**Table 2:** Antibodies

Antigen	Clone/product name	Dilution	Host species	Source	Catalog #
pSRRM2	SC-35	1:200	Mouse	SigmaAldrich	S4045
pTau Thr231	AT180	1:200	Mouse	ThermoScientific (Rockford, IL, USA)	MN1040
NeuN	A60	1:400	mouse	Millipore	MAB377
Tau (Total)	SP70 pAb	1:1,000	Rabbit	Rockland Immunochemicals Inc. (Limerick, PA, USA)	200-C01-B33
pTau Ser202	CP13 mAb	1:500	Mouse	Peter Davies (Litwin-Zucker Research Center for the Study of Alzheimer's Disease, The Feinstein Institute of Medical Research, Northwell Health, Manhasset, NY, USA)	N/A
pTau Ser396/Ser404	PHF-1 mAb	1:2,000	Mouse		
pTau Ser422	EPR2866 mAb	1:500	Rabbit	Abcam (Cambridge, UK)	ab79415
pTau Thr181	AT270 mAb	1:15,000	Mouse	ThermoFisher Scientific (Waltham, MA, USA)	MN1050
Tau (Total)	K9JA (DAKO) pAb	1:250	Rabbit	Agilent Technologies, Inc. (Santa Clara, CA, USA)	A002401-2
2° Ab Mouse	Horseradish Peroxidase α-Ms IgG (H + L)	1:5,000	Goat	Jackson Immunoresearch (West Grove, PA, USA)	115-035-146
2° Ab Rabbit	Horseradish Peroxidase α-Rb IgG (H + L)	1:5,000	Goat	Jackson Immunoresearch (West Grove, PA, USA)	111-035-144
2° Ab Mouse	Alexa Fluor® 568 Goat α-Ms IgG (H + L)	1:1,000	Goat	Invitrogen (Carlsbad, CA, USA)	A-11004
2° Ab Rabbit	Alexa Fluor® 647 Goat α-Rb IgG (H + L)	1:1,000	Goat	Invitrogen (Carlsbad, CA, USA)	A-21245

### Tau pathology drives mis-localization and pathological deposition of phospho-SRRM2 into tau lesions

**Fig. 2:**
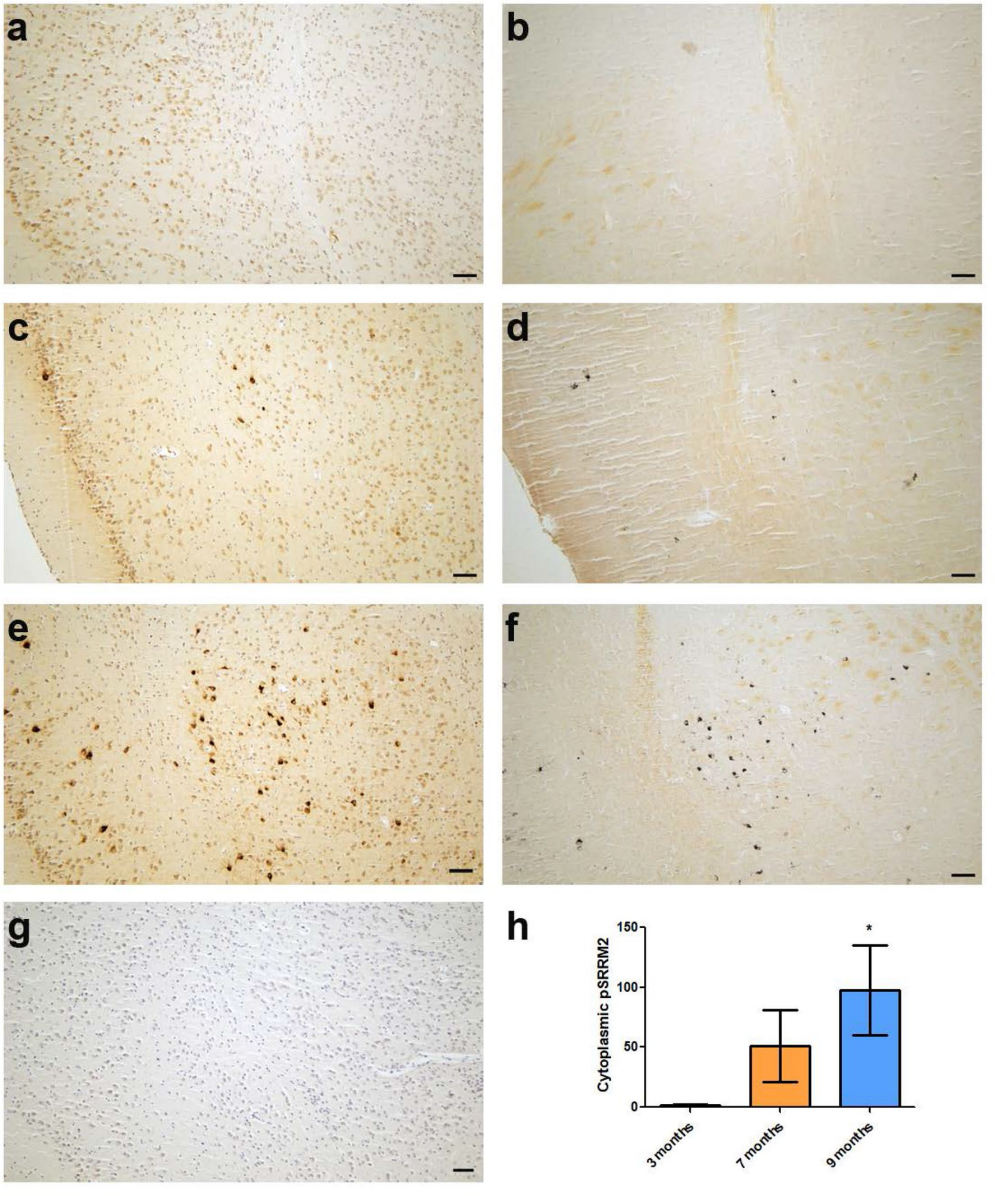
Abnormal pSRRM2 mis-localization occurs in a transgenic mouse model of tauopathy. Immunostaining with SC-35 antibody (**a**, **c**, **e**, **g**) demonstrates that pSRRM2 is mis-localized to the neuronal cytoplasm in brain regions of PS19 mice where Gallyas positive NFTs (**b**, **d**, **f**) are prevalent. At three months, pSRRM2 appears as diffuse mild immunoreactivity in the cytoplasm (**a**) and there are no tangles (**b**). At seven months, a small number of Gallyas positive NFTs are present and neurons with robust cytoplasmic distribution are detectable in these same regions (**c**, **d**). By nine months, PS19 mice with robust tangle burden display abnormal pSRRM2 cytoplasmic localization in a large number of neurons (**e**, **f** and quantified in **h**). In contrast, pSRRM2 is not detectable in a nine-month-old non-Tg mouse brain (**g**). (*p = 0.04 by two-tailed Student’s t test) Scale bars, 100 µm

Because pSRRM2 mis-localization occurs in the same brain regions as neurofibrillary degeneration, we hypothesized that pathological tau deposition provokes ectopic pSRRM2 accumulation in the neuronal soma. To test this idea, we examined the pattern of pSRRM2 staining in the brains of PS19 tau transgenic mice. This well characterized mouse model of tauopathy exhibits high level expression of the FTLD P301S mutant human tau in degenerating neurons [23]. While SRRM2 is well conserved between humans and mice [29], phosphorylation of SRRM2 is complex with potential variation between mice and humans [22, 26]. In aged PS19 mice following onset of neurofibrillary tangle deposition, pSRRM2 mis-localization becomes evident and is similar to the aberrant cytoplasmic accumulation we observed in AD brain (Fig. 2a–f, h). In non-Tg mice, mAb clone SC-35 does not detect any pSRRM2 protein (Fig. 2 g) and in 3-month-old PS19 mice pSRRM2 appears as diffuse mild immunoreactivity in the cytoplasm (Fig. 2a) and there are no tangles (Fig. 2b). We find robust accumulation of cytoplasmically mis-localized pSRRM2 (Fig. 2c) in a subset of neurons and detect sparse NFTs in 7-month-old PS19 mice (Fig. 2d). By nine months of age, PS19 mice exhibit significant pSRRM2 cytoplasmic accumulation in a substantial fraction of neurons in these same regions (Fig. 2e) and have accumulated substantial hippocampal and cortical NFTs (Fig. 2f) and begun to exhibit frank neurodegeneration. To validate these findings in a mouse model of tauopathy driven by wild type human tau expression, we examined pSRRM2 accumulation in the Tau4Rtg2652 mouse line, a model with early stage tau pathology, including phosphorylated tau, but without neurofibrillary degeneration. The Tau4Rtg2652 mice exhibit a milder but disrupted nuclear speckle staining pattern for pSRRM2 (Additional file 1: Fig S1). Because expression of the tau transgene drives the primary insult in tau transgenic mice, these findings suggest that pSRRM2 mis-localization to the cytoplasm occurs in neurons as a result of pathological tau accumulation leading to neurodegeneration accompanied by mis-localization of pSRRM2. Further, these data suggest a hypothesis where cytoplasmic pSRRM2 marks a new subtype of neuropathological lesion associated with pathological tau in AD.

### SRRM2 + lesions co-localize with pathological tau in the cytoplasm and pSRRM2 becomes depleted from the nucleus

**Fig. 3:**
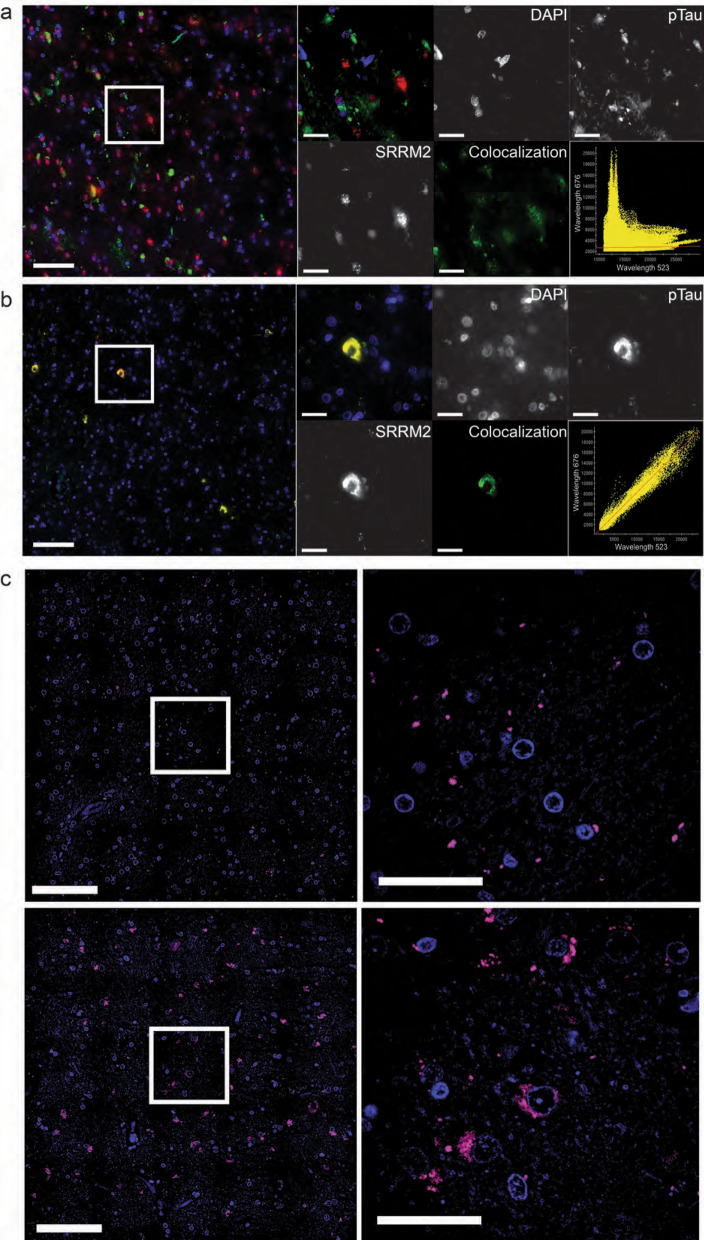
pSRRM2 co-localizes with pathological tau in AD. **a** pSRRM2 (Red) is localized to the nucleus and pTau (pS422-Green) is cytoplasmic in cognitively normal elderly controls; visible overlapping signal is visualized as yellow (Pearson Coefficient of Colocalization = 0.017), (Panel image scale bar = 100um; inset scale bar = 25um). **b** pSRRM2 (Red) relocalizes to the cytoplasm and colocalizes with pTau (Green), as visualized in yellow in AD patients (Pearson Coefficient of Colocalization = 0.99). (Panel image scale bar = 100um; inset scale bar = 10um). **c** Proximity Ligation Assay imaging between pSRRM2 and pTau. Top: Panel image and inset imaging of PLA colocalization in a cognitively normal elderly control donor shows minimal cytoplasmic colocalization (Panel image scale bar = 100um; inset scale bar = 10um). Bottom: Panel image and inset imaging of PLA colocalization in an AD donor show significant colocalization in the cytoplasm (Panel image scale bar = 100um; inset scale bar = 10um)

To test the hypothesis that pSRRM2 + lesions overlap with pathological tau, we conducted co-immunofluorescent staining with antibodies against tau and pSRRM2 to fluorescently label pSRRM2 + and Tau + deposits in AD cases. From our co-labeling studies, we observe no obvious co-localization between SC-35 staining of pSRRM2 and pTau in cognitively normal controls (Fig. 3a). However, we observe strong co-localization between pSRRM2 and pTau in AD cases, with Pearson coefficient of colocalization (PCC) of 0.99 (Fig. 3b). In addition, pSRRM2 becomes depleted from the nucleus in cases with cytoplasmic pSRRM + deposits both in tangle bearing and non-tangle bearing neurons. Further we demonstrated by proximity ligation assay that the tau positive lesions remain in close proximity to pSRRM2 + lesions localized in the cytoplasm of cortical neurons predominantly in AD patient brains but not age matched control brains (Fig. 3c).

### SRRM2 mis-localization overlaps with MSUT2 depletion in AD

We previously showed that the nuclear speckle protein MSUT2 and its binding partner PABPN1 reciprocally influence vulnerability to tauopathy, and both become depleted in more severe AD cases [19]. To assess the interaction between cytoplasmic accumulation of pSRRM2 and MSUT2 in AD, we immunostained the frontal cortex of our AD cohort for MSUT2 (Table 3). All AD cases with predominately nuclear pSRRM2 (n = 9) had intact MSUT2 protein levels and milder tauopathy. In contrast, of our 18 AD cases with substantial pSRRM2 cytoplasmic accumulation in the frontal cortex, 14 (78%) had depleted MSUT2 and 17 (94%) exhibited robust or moderate phospho-tau pathology. These data support the idea that MSUT2 depletion contributes to pSRRM2/Tau + co-pathology in AD and suggest nuclear speckle disruption occurs as a cellular feature of AD.

**Table 3:** Case IHC data summary

Case #	Dx	pSRRM2	MSUT2	AT180
10	AD	Cytoplasmic	Depleted	+++
6	AD	Cytoplasmic	Depleted	+++
8	AD	Cytoplasmic	Depleted	+++
9	AD	Cytoplasmic	Depleted	+++
16	AD	Cytoplasmic	Positive	+++
5	AD	Cytoplasmic	Depleted	+++
1	AD	Cytoplasmic	Depleted	+++
2	AD	Cytoplasmic	Depleted	+++
3	AD	Cytoplasmic	Depleted	+++
7	AD	Cytoplasmic	Depleted	++
13	AD	Cytoplasmic	Positive	++
11	AD	Cytoplasmic	Depleted	++
4	AD	Cytoplasmic	Depleted	++
12	AD	Cytoplasmic	Positive	++
18	AD	Mixed	Depleted	+++
17	AD	Mixed	Depleted	+++
20	AD	Mixed	Positive	++
19	AD	Mixed	Depleted	+
28	AD	Nuclear	Positive	++
23	AD	Nuclear	Positive	++
29	AD	Nuclear	Positive	+
24	AD	Nuclear	Positive	+
22	AD	Nuclear	Positive	+
21	AD	Nuclear	Positive	+
26	AD	Nuclear	Positive	+
27	AD	Nuclear	Positive	+

+, mild; ++, moderate; +++, robust AT180 p-tau accumulation

### Pathological accumulation of cytoplasmic pSRRM2 coincides with tauopathy severity in Alzheimer’s Disease

**Fig. 4:**
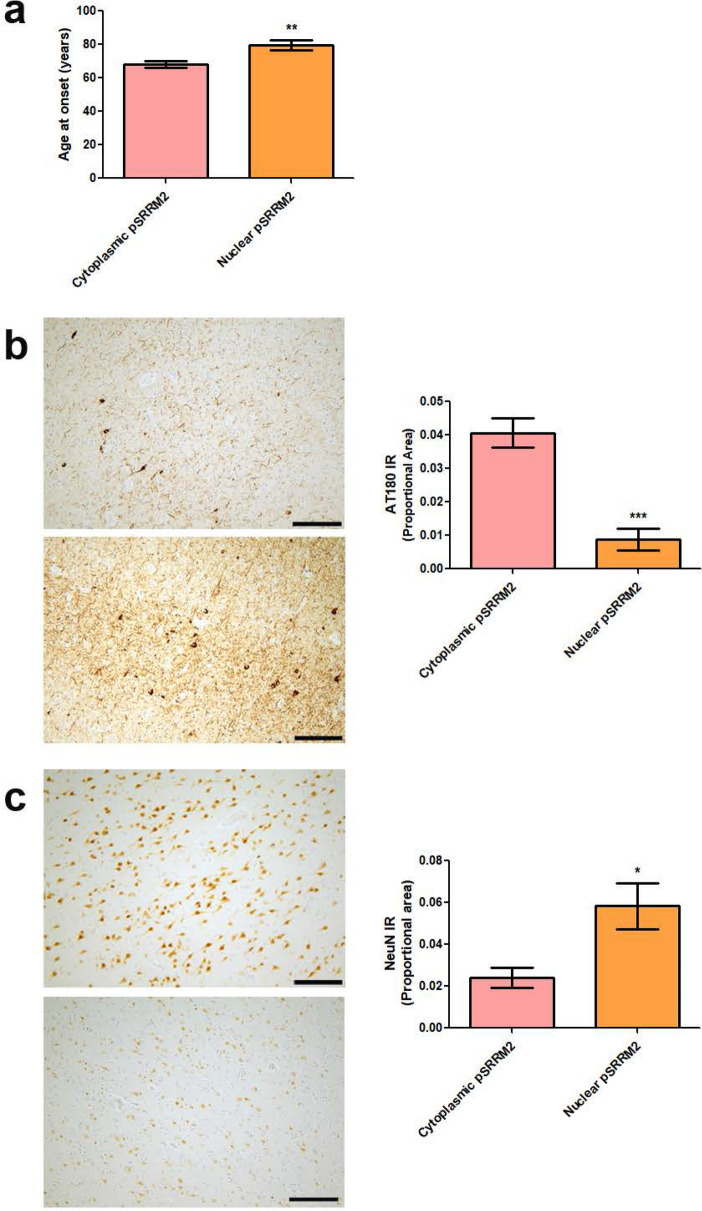
pSRRM2 mis-localization in AD associates with disease severity. Cytoplasmic pSRRM2 accumulation in AD brain is associated with a more aggressive disease progression. **a** AD donors with predominately cytoplasmic pSRRM2 in postmortem frontal cortex (n = 20) had an earlier age of disease onset compared to those with primarily nuclear pSRRM2 distribution (n = 9) (**p = 0.0042 by two-tailed Student’s t test). **b** Representative brain sections from frontal cortex of an AD donor with normal nuclear pSRRM2 distribution (top) compared to an AD donor with abnormal cytoplasmic accumulation of pSRRM2 (bottom) stained with the anti-phosphorylated tau antibody AT180. Brain tissue from cases with cytoplasmic pSRRM2 exhibited more pathological tau. Densitometry analysis of AT180-positive reactivity in AD cases with nuclear (n = 8) or cytoplasmic (n = 19) pSRRM2 (***p = 0.0002 by two-tailed Student’s t test). **c** Representative brain sections from postmortem brain frontal cortex of an AD donor with normal nuclear pSRRM2 distribution (top) compared to an AD donor with abnormal cytoplasmic accumulation of pSRRM2 (bottom) stained with the neuronal marker NeuN. Brain tissue from cases with cytoplasmic pSRRM2 exhibited decreased NeuN immunoreactivity (indicative of more neuronal loss). Densitometry analysis of NeuN-positive reactivity in AD cases with nuclear (n = 9) or cytoplasmic (n = 18) pSRRM2 (**p = 0.0025 by two-tailed Student’s t test). Scale bars, 250 µm

Given the potential involvement of nuclear speckle components in tauopathy and co-deposition of pSRRM2 with tau in pathological lesions in AD, we examined the relationship between mis-localization of pSRRM2 and disease severity. First, we tested for associations between pSRRM2 and clinical AD manifestation by comparing age at onset and the presence of pSRRM2 within the neuronal soma in AD frontal cortex where we noted cytoplasmic pSRRM2 in th 72% of AD cases. AD cases bearing cytoplasmic pSRRM2 exhibited a relatively younger age of AD onset (11 years earlier) as compared to cases lacking pSRRM2 mis-localization (Fig. 4a). Next, we examined the relationship between pSRRM2 mis-localization and frontal cortical pTau burden in AD, where we observed a significant increase in pTau abundance in cases with cytoplasmic mis-localization of pSRRM2 compared to cases with normal nuclear distribution (Fig. 4b). Finally, we assessed neurodegeneration through NeuN immunohistochemistry, where we observed increased neuronal loss in AD cases with aberrant cytoplasmic pSRRM2 (Fig. 4c). Taken together, these results demonstrate that cytoplasmic distribution of pSRRM2 appears to coincide with exacerbated AD clinical onset and neuropathological severity.

## Discussion

### Pathological tau drives nuclear speckle scaffold protein mislocalization to the cytoplasm

**Fig. 5:**
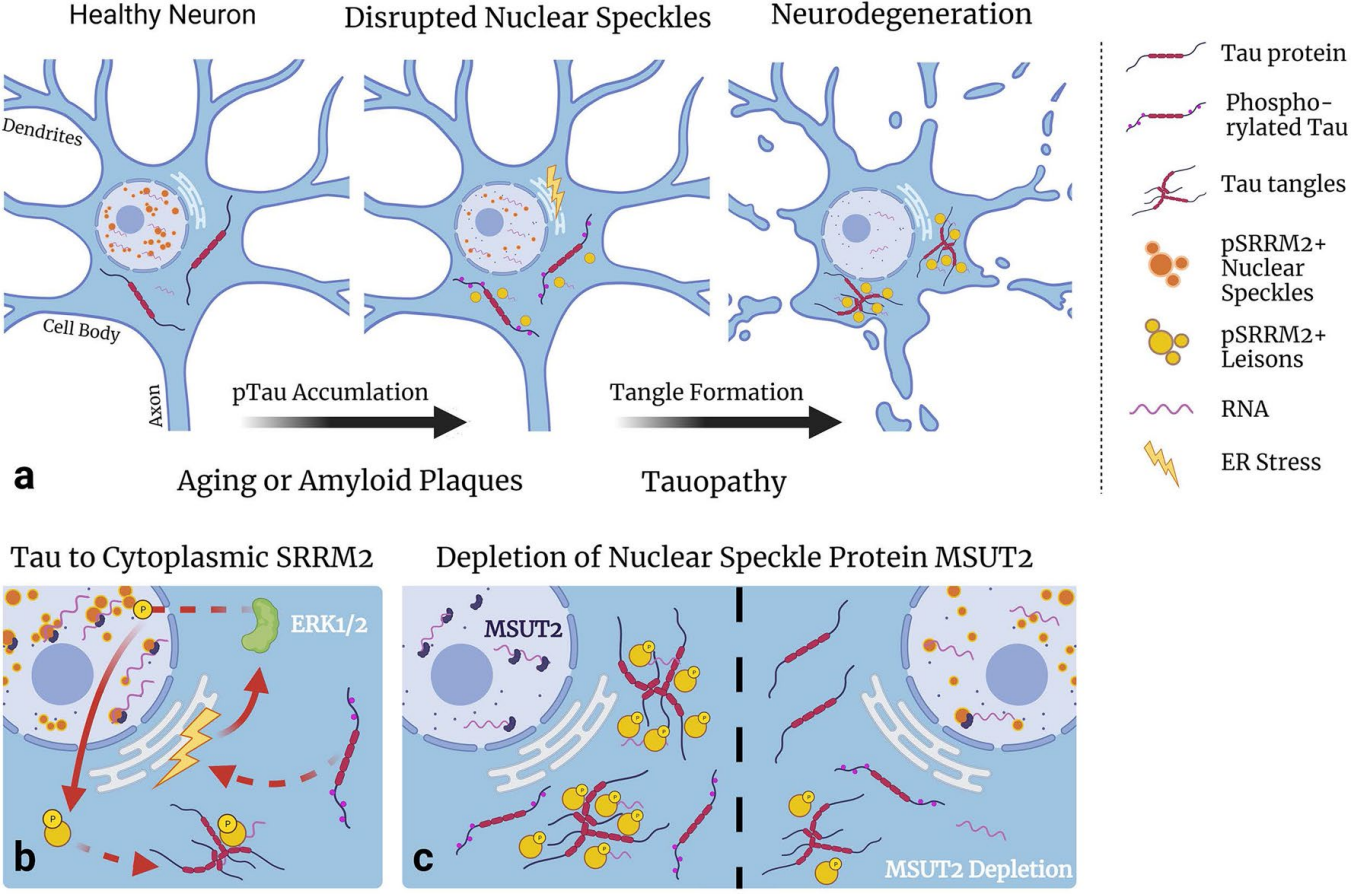
Nuclear Speckle disruption occurs as a result of pathological tau in AD. **a** Changes associated with aging, including decreased proteostasis, increased neuroinflammation, and metabolic challenge, contribute to the initiation of amyloid plaque deposition. Plaques have been shown to trigger a tauopathy cascade. Here we demonstrate that pathological tau deposition coincides with depletion of nuclear speckle scaffold protein SRRM2 from the nucleus. Nuclear SRRM2 function within nuclear speckles is required for appropriate mRNA processing. Inefficient mRNA processing causes neurodegeneration. **b** Phosphorylation of SRRM2 at pS1068 drives SRRM2 transit from the nucleus to cytoplasm and we propose that pathological tau provokes phosphorylation and mislocalization of SRRM2 through an ER stress and ERK1/2 mediated pathway which leads to neurodegeneration. **c** Modulation of nuclear speckle content via loss of regulatory RNA binding proteins resident in nuclear speckles such as SUT-1 or SUT-2/MSUT2 can fully rescue tauopathy phenotypes. We propose that the *sut* RNA binding protein pathway modulates tauopathy by restoring nuclear speckle function in the face of pathological tau

We observed ectopic accumulation of the nuclear speckle scaffold protein SRRM2 in the cytoplasm in AD, and pSRRM2 + deposits overlap with the neurofibrillary tangle pathology in AD. To investigate the relationship between pathological tau and ectopic SRRM2, we employed robust animal models of pathological tau deposition and found that pSRRM2 became progressively recruited to the cytoplasm as pathological tau deposition increased. In addition, pathological tau and pSRRM2 + deposits co-localize within the neuronal soma and remain in close proximity within tangle bearing neurons. Taken together, these findings support a tauopathy cascade model whereby Aβ, aging, or some other stimulus triggers pathological tau deposition in the cytoplasm, which recruits pSRRM2 to the fibrillar tau deposits leading to dysfunction of nuclear speckles (Fig. 5a).

### RNA-binding proteins modulate tauopathy

We previously identified RNA binding proteins that reside in nuclear speckles, including *sut-1*, *sut-2/MSUT2*, and *parn-2/TOE1*. Examination of genes required for tau pathology-driven neurodegeneration in *C. elegans* uncovered *su*ppressor of *t*auopathy 1 (*sut-1),* a nuclear speckle resident protein whose loss of function prevents tau-mediated neurodegeneration; *sut-1* functions in speckles and plays a role in *C. elegans* trans-splicing [14]. The same genetic approach uncovered *sut-2* as a genetic suppressor of tauopathy [16], and subsequent translational studies revealed that the mammalian homolog of *sut-2* (*MSUT2*) also resides in nuclear speckles and plays a critical role in tau aggregation and neurodegeneration in mice, human cells, and AD brain tissue [19, 25]. Recent studies of other nuclear speckle resident proteins revealed that the nuclear speckle resident RNA nucleases *parn-1*/PARN and *parn-2*/TOE1 play distinct roles in tau toxicity [15]. Contemporaneous work from other laboratories has also implicated RNA-binding proteins in tauopathy, including Musashi (MSI) and T-cell intracellular antigen 1 (TIA1); while they have divergent RNA-binding functions, both TIA1 and MSI co-localize with tau-containing cytoplasmic lesions and modulate tau aggregation and concomitant tauopathy phenotypes in model systems [30–33].

### SRRM2 phosphorylation and mis-localization occurs in AD

Tanaka and colleagues demonstrated that abnormally localized SRRM2 occurs in both AD and animal models of Aβ deposition, driving aberrant splicing [22]. They further reported that phosphorylation of SRRM2 at pS1068 stimulates SRRM2 transit from the nucleus to cytoplasm, and hypothesized that ER stress activation of ERK1/2 drives pSRRM2 phosphorylation [22]. Here, we show that pathological tau, in the absence of Aβ, can provoke pSRRM2 accumulation in the cytoplasm in mouse models, consistent with our observation that abnormal pSRRM2 deposition coincides with severity of AD tau pathology. Recent work has also demonstrated that pathological tau drives ER stress in both animal models [34–36] and in AD [37–40]. We hypothesize a cascade where pathological tau provokes pSRRM2 mislocalization through an ER stress and ERK1/2 mediated pathway resulting in aberrant splicing and ultimately neurodegeneration (Fig. 5b).

### Tau aggregates as RNA–protein assemblies associated with nuclear speckles

Several studies have shown that tau immunopurifies with several RNA-binding proteins [41, 42], and recent work has nominated nuclear speckles as a participant in tauopathies through the scaffold protein SRRM2. Lester and colleagues showed that tau deposits purified from model systems, including mice and human cells, contain many RNAs and are highly enriched for small nuclear and small nucleolar RNAs (snRNAs and snoRNAs) [7]. These findings are consistent with previous work showing that tau aggregates in brain are typically RNA-positive [43], and suggest pathological properties for nuclear tau [42, 44–46], as Lester and colleagues speculate pathological tau may be seeded by RNA into nuclear aggregates in human tauopathy disorders. In model systems this may be particularly relevant, as tau truncated species accumulate to high levels in many models, and tau fragments readily relocalize to the nucleus in some cellular systems [47]. Investigation of a small number of human tauopathy cases demonstrated mislocalization of SRRM2 in AD, but did not document the presence of nuclear tau/RNA assemblies [7]. Here, we confirm nuclear clearance and cytoplasmic accumulation of pSRRM2 in a larger AD cohort, and demonstrate variability in pSRRM2 related pathologic changes in the frontal cortex of AD cases. In sum, these studies highlight the dysfunction of nuclear speckles in AD. To further understand the mechanistic disease relevance of nuclear speckle disruption in AD, transcriptomic and proteomic characterization of pSRRM2 + cytoplasmic deposits in AD should be prioritized in future work, and comparison of SRRM2 + deposits between AD brain and model systems may be critical to understand possible disease mechanisms.

### Tau mediates neurodegeneration through nuclear RNA processing defects

Multiple studies have demonstrated that tau neuropathology drives neurodegeneration by causing dysfunction of nuclear RNA processing events (reviewed in [48]). Recently, tau aggregates have been shown to disrupt the nuclear pore complex, blocking export of mRNAs via nuclear depletion and co-aggregation of tau with the disordered region of Nup98 [49]. This molecular phenotype is reminiscent of the findings reported here for pSRRM2, which is co-deposited with pathological tau. Subsequent related work in model systems has shown clear evidence for mRNA accumulation within nuclear invaginations caused by pathological tau, further supporting disruption of RNA trafficking as a critical pathological consequence of tau aggregation [6]. Other studies have implicated defects in mRNA splicing as a critical nuclear process disrupted in AD specifically nominating disruption of U1 snRNP via mislocalization of the RNA binding protein U1-70 K [42, 50, 51]. Further, tau mediated spliceosome dysfunction has been hypothesized as a trigger of cryptic RNA splicing and consequent neurodegeneration in AD [12].

### Tau mediated neurodegeneration can be modulated by altering nuclear speckle contents

A recurring theme in tau mediated neurodegeneration is the depletion of an essential RNA-binding protein from the nucleus as a result of co-aggregation with cytoplasmic pathological tau. We reiterate this has been shown to occur for pSRRM2 (this study & [7]), Nup98 [49], and U1-70 k [42, 50, 51]; the loss of function for any one of these three critical proteins likely dooms a neuron to degeneration. However, previous genetic studies have demonstrated rescue of tauopathy phenotypes by loss of function mutations in RNA-binding proteins. Modulation of nuclear speckle content via loss of regulatory RNA-binding proteins resident in nuclear speckles, such as SUT-1 or SUT-2/MSUT2, can fully rescue tauopathy phenotypes in model systems including tau transgenic *C. elegans,* human cells and mouse brains [16, 19, 25]. We hypothesize that the *sut* RNA-binding protein pathway modulates tauopathy by restoring nuclear speckle function in the face of pathological tau (Fig. 5c).

The findings of our work, combined with the evidence from the literature, support the hypothesis that changes in the content of nuclear speckles contributes to tauopathy mediated neurodegeneration in AD. Tau-mediated neurodegeneration can be rescued in animal models by altering the content of nuclear speckles, for instance by targeting MSUT2 or related proteins [19]. Examination of the transcriptome and proteome of SRRM2 + /tau deposits and nuclear speckles in AD remain important future directions for our work investigating the molecular mechanisms of tauopathy disorders and will inform future molecular dissection of tau pathobiology.

## Supplementary Information

**Additional file 1: Fig. 1.** Tau4Rtg2652 mice exhibit a milder but disrupted nuclear speckle staining pattern for pSRRM2. Immunostaining with SC-35 antibody demonstrates that pSRRM2 is mis-localized in hippocampal CA3 neurons of a four-month-old Tau4Rtg2652 mouse (b) and appears as diffuse cytoplasmic staining. No immunoreactivity is detectable in a four month old non-Tg mouse brain (a). Scale bars, 50 µm.

## Data Availability

The datasets generated and analyzed during this study are included in this published article and its supplementary information files, or are available from the corresponding author on reasonable request.
