# Supplementary material for: Pathological tau drives ectopic nuclear speckle scaffold protein SRRM2 accumulation in neuron cytoplasm in Alzheimer’s disease
Source: Acta Neuropathol Commun. 2021 Jun 29;9:117. doi: 10.1186/s40478-021-01219-1 (PMC8243890; doi:10.1186/s40478-021-01219-1)
Supplement: Supplementary file 1 [file 40478_2021_1219_MOESM1_ESM.docx]

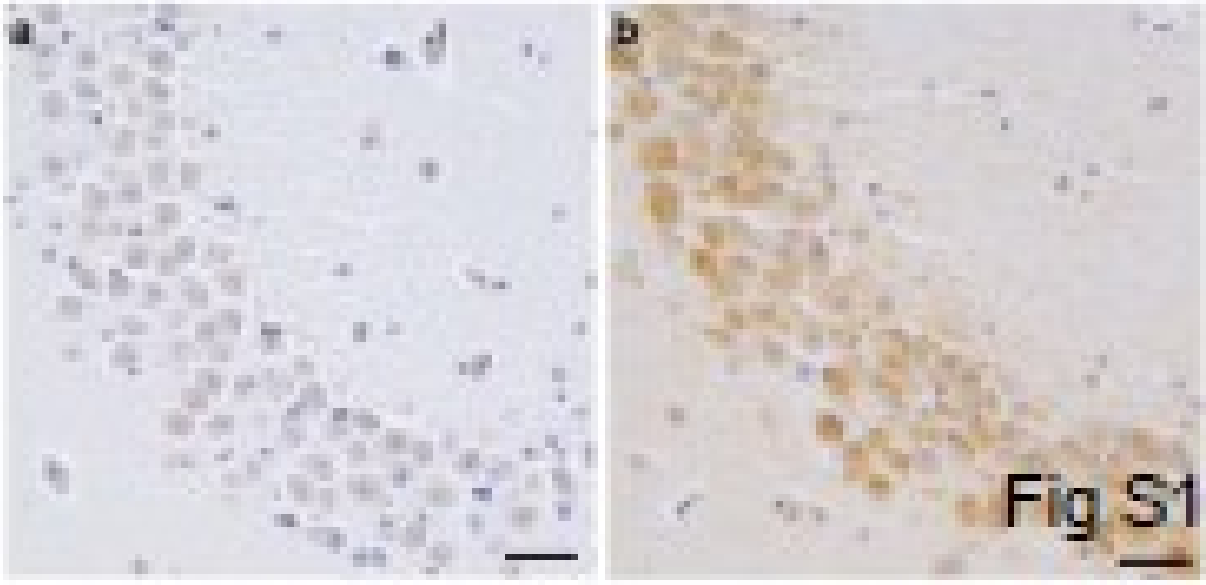

Fig S1
